# Supplementary material for: Are carbon emissions trading and green financial instruments synergistic? -Comprehensive quantitative research based on content analysis
Source: PLoS One. 2024 Mar 7;19(3):e0298601. doi: 10.1371/journal.pone.0298601 (PMC10919601; doi:10.1371/journal.pone.0298601)
Supplement: S1 Data — (ZIP) [file pone.0298601.s001.zip › Data/地级市Z_F工作报告.zip!/║╙─╧/╓ú╓▌╩╨/└┤╫╘╣1⁄2╓┌║┼í╛┬φ┐╦╩2╛▌═°í┐_2010.docx]

各位代表：

现在，我代表市人民政府，向大会作工作报告，请予审议，并请各位政协委员提出意见。

一、2009年工作回顾

2009年是新世纪以来我市经济发展最为困难的一年。市人民政府在省委、省政府和市委的正确领导下，团结带领全市人民，深入学习实践科学发展观，坚决执行中央、省扩内需、保增长的一系列政策措施，以跨越式发展十项重点工程为抓手，坚定信心、科学应对，凝心聚力、克难攻坚，决战二季度、大干三季度、决胜四季度，在应对金融危机中取得了保增长、保态势、保民生、保稳定的重大成效，较好地完成了市十三届人大一次会议确定的各项目标任务。

初步统计，全市实现生产总值3300亿元，增长12%；地方财政总收入达到521.7亿元，增长12.3%，其中一般预算收入达到301.9亿元，增长15.9%；全社会固定资产投资完成2289.1亿元，增长29.1%；社会消费品零售总额完成1434.8亿元，增长18.9%；城镇居民人均可支配收入达到17417元，农民人均纯收入达到8121元，分别实际增长9.5%和7.8%。

（一）战危机保增长成效明显。全面落实中央、省战危机、保增长的一系列重大决策部署，结合我市实际，提出了“以建补工、以三补二、以内补外、以轻补重、以高补低”的思路，出台了《关于应对金融危机保持经济平稳较快增长的意见》，先后召开4次经济运行工作会议进行安排部署，在建立中小企业信用担保体系、扶持汽车和电解铝行业稳定生产、促进房地产业健康发展等方面采取了一系列政策措施，有效缓解了金融危机对我市经济的冲击，遏制了经济下滑势头。贯彻落实积极的财政政策，全年一般预算支出353亿元，增长22.3%；安排产业发展引导资金17亿元，其中新增支持汽车产业发展、电解铝储备、信用担保体系建设等资金10.5亿元；争取地方政府债券资金12.5亿元，全年金融机构新增贷款1309.9亿元。坚定不移地实施跨越式发展战略，制定了新三年行动计划，确定了十项重点工程356个项目。强化投资对经济增长的拉动作用，城镇固定资产投资完成2002.2亿元，增长31.6%。鼓励和扩大消费需求，及时出台了搞活流通、扩大消费的意见，制定了拉动城乡消费、优化商贸网络布局、促进消费升级等17条具体措施，安排了商贸流通业发展专项资金。深入开展企业服务年活动，把1312家企业作为重点服务对象，向170家重点服务企业派驻78个工作组；结构性减税30亿元，支持企业扩大投资；组织银企合作和产销对接，帮助企业克服生产经营困难。

（二）工业经济运行逐步好转。落实扶持政策，强化经济运行监测，突出项目建设，大力实施扶优扶强、产业集聚区建设和科技自主创新工程，工业经济运行企稳回升。规模以上工业完成增加值1332.5亿元，增速由一季度的－5.3%提高到四季度的28.4%，全年增长11.2%；实现销售收入4727.4亿元，实现利润501.1亿元，分别增长9.3%和12.4%。落实国家、省十大产业振兴和调整规划，积极编制汽车工业、纺织服装、电子商务、铝工业、食品工业、电子信息等专项规划，制定了企业间协作联合、兼并重组、拓宽融资渠道等应对措施。充分发挥投资对工业增长的拉动作用，工业投资完成926.4亿元，有60个项目投入超亿元，郑煤机高端液压支架生产基地、海马（郑州）年产30万台节能发动机等260个重点项目开工建设。扶优扶强工程加快推进，57家重点企业实现销售收入1213亿元。产业集聚区建设不断加快，加大了规划编制和基础设施建设力度，加快投融资平台建设，39个重点产业集聚区实现销售收入2845.7亿元，增长20.9%。高新技术产业发展迅速，新增市级以上企业技术中心118个、工程研究中心40个，高新技术产业实现产值1683亿元，增长22%，我市被认定为国家科技进步示范城市，国家知识产权示范城市创建工作通过验收。

（三）服务业发展实现新突破。壮大批零商贸、住宿餐饮等传统服务业，大力发展物流、会展、金融、文化旅游等现代服务业。全年第三产业增加值达到1400亿元，增长13%。商贸流通业发展迅速，编制了《郑州市现代化商贸城建设总体规划》和《郑州市商业网点规划》，有10家商贸流通企业年销售额超10亿元。“万村千乡”市场工程积极推进，“家电下乡”工作深入开展，新农村现代流通网络工程加快实施。区域性金融中心建设取得新进展，郑州市商业银行更名为郑州银行，入驻郑州新区和签订入驻协议的金融机构累计达到53家；郑州商品交易所成交金额19.1万亿元，增长22.8%。物流枢纽建设不断加快，编制了《郑州国际物流中心战略规划》，努力打造中西部领先的综合内陆无水港、重要的航空货运基地、辐射全国的区域分拨中心；铁路集装箱中心站场站工程等一批重大项目顺利竣工，国家干线公路物流港、河南进口物资公共保税中心（B型）等项目进展顺利。会展经济繁荣发展，全年举办全国药品交易会、全国汽车配件交易会、全国糖酒商品交易会等大型展会84场，累计办展面积124万平方米，拉动经济增长100亿元以上。文化旅游业加快推进，市图书馆新馆、商城遗址公园、国家动漫产业发展基地（河南基地）等项目进展顺利；发放1亿元旅游消费券，接待国内外游客4167.7万人次，旅游业总收入达到398.6亿元，增长16.6%；成功举办了己丑年黄帝故里拜祖大典；黄河风景名胜区被评为国家级风景名胜区。房地产销售形势较好，全市商品房销售面积1198.9万平方米，增长62.7%；销售金额514.8亿元，增长77.9%。中介咨询、动漫创意、服务外包等新兴服务业快速发展。

（四）农业和农村经济稳步发展。大力发展现代农业，加强农村基础设施建设，扎实推进新农村建设。全年粮食总产量达到166.1万吨，再创历史新高。农业产业化经营进程加快，市级以上农业龙头企业达到289家，实现销售收入300亿元；新建现代农业示范园区2个，新发展农民专业合作社160个。农业综合开发稳步推进，改造中低产田10.5万亩。农村基础设施建设得到加强，新修改造农村公路1007公里； 新增有效灌溉面积34万亩，治理水土流失面积101平方公里，解决了37万人的饮水安全问题；新增农村户用沼气3万座、大中型沼气工程61处，全市已有20万农村家庭用上干净高效的户用沼气。新农村建设稳步推进，102个建设项目完成投资55.1亿元。

（五）郑州新区建设全面启动。紧紧抓住省委、省政府实施郑汴新区建设的重大战略机遇，把郑州新区作为全市经济社会发展的“一号工程”，统一思想，强力推进。重大基础设施建设初见成效，高速铁路综合交通枢纽站、机场至民权高速公路、四港联动大道等一批项目相继开工，郑州东500千伏输变电工程、陈三桥污水处理厂一期工程基本建成，郑新黄河公铁两用大桥、国际航空港配套工程等项目顺利推进。重大产业项目加快实施，68项重大产业项目累计完成投资80亿元，其中，海马（郑州）轿车生产基地微轿生产线等16个项目已竣工投产或试生产，郑州日产第二工厂等30个在建项目取得实质性进展。招商引资取得重大突破，签约项目投资总额共523亿元。投融资工作扎实开展，成立了郑州新区建设开发投资总公司，企业债券发行进展顺利。

（六）城市综合承载能力进一步提升。继续加大基础设施建设力度，城镇化步伐不断加快，城镇化率达到63.3%。着力构建城市综合交通体系，配合做好石武、郑西、郑徐铁路客运专线建设工作，郑州至焦作、郑州至开封、郑州至机场等城际轨道交通工程开工建设。市政重点工程进展顺利，65个城建项目累计完成投资100亿元，是近年来投资规模最大的一年。南三环—嵩山路立交、天河北路等一批市政交通工程建成启用，京广路—沙口路高架快速通道、中心区铁路跨线桥、火车站西出口站前广场等工程加快推进。地铁1号线开工建设，文化路—东风路等8座下穿式隧道和8座人行过街天桥加快建设。改造背街小巷100条，完成6个积水点改造，完成雨污水改造工程41项，铺设管涵14公里。五龙口污水处理厂二期等项目建成试运行。快速公交通车运营。市区新建公园3个、游园16个，植物园一期竣工开园，新增绿地561万平方米。供水、供电、供气和防洪等设施建设进一步加强。城中村和旧城改造稳步推进，城市功能进一步完善。深入开展市容市貌、占道经营、早夜市、建筑垃圾运输、户外广告等专项整治活动，全市市容管理秩序逐步好转。城市数字化管理系统全面启用。卫星城和小城镇建设步伐加快。城镇体系规划编制工作全面展开，《数字郑州总体规划》通过评审。

（七）改革开放继续深化。稳步推进国有企业战略重组和上市工作，白鸽集团等企业改制工作进展顺利，华润集团控股郑州燃气，商业大厦重组正式启动。投融资体制进一步理顺，多主体、多渠道、多方式融资316亿元。全市新增上市企业4家，总数达到25家。同时，全市集体林权制度改革主体任务基本完成，政府机构改革全面启动，事业单位分类改革、医药卫生体制改革、文化体制改革和农村综合配套改革正在抓紧推进。营造有利于民营经济发展的市场环境，大力支持并积极引导民营经济加快发展。抓住全球资本加快流动和沿海地区产业向中西部快速转移的重大机遇，继续扩大对外开放，加大招商引资力度，全面开展 “南下北上”大招商活动，积极参加第四届中博会、中原文化澳洲行、台湾经贸文化交流等活动，特别是加大与中粮、中兵、中船等央企的战略合作，通过“大招商、招大商”，全年实际利用外资16.2亿美元，引进域外境内资金609.3亿元。支持外经外贸企业加快发展，及时出台了加快外经外贸发展的意见和配套办法，支持中小企业开拓国际市场，帮助企业扩大出口规模。

（八）节能减排和生态建设深入推进。积极推动重点领域节能减排，加快推进耗能企业技术改造，大力发展循环经济，全年单位生产总值能耗下降5.8%，二氧化硫排放量和化学需氧量分别控制在14.9万吨和5.42万吨。生态环境质量明显改善，森林生态城建设成效明显，提前4年超额完成100万亩的栽植任务；建成2个林业生态县、100个林业生态村和8个林业生态模范村。城区环境空气质量优良天数达到322天，全市饮用水源地水质达标率保持100％。生态水系建设取得阶段性成果，七里河、魏河、十七里河、十八里河、索须河二期、南部河道输水等主要河流河道治理工程全部完工。第二届中国绿化博览会筹备工作加快推进，绿博园基础设施工程基本完工。南水北调工程郑州段全面开工，移民安置工作进展顺利。

（九）人民生活不断改善。加大对民生领域的投入，向人民群众承诺的“十大实事”全部完成。全年用于民生方面的财政支出达到171.6亿元，增长28.3%，占一般预算支出的48.6%。实施积极的就业政策，新增就业再就业12.6万人，农村劳动力转移就业12.5万人。企业职工基本养老保险新增参保10万人，22.7万企业离退休人员每月人均增加养老金123.8元，城乡基本养老保险参保人数达到43.2万。城镇职工基本医疗保险、城镇居民基本医疗保险和新型农村合作医疗参保人数分别达到83万、125万和410万，将全市国有和集体破产、困难企业退休人员全部纳入基本医疗保险；新农合报销比例提高10%，报销封顶线由3万元增至5万元。为城乡低保人员发放了生活救助物资，在全省率先建立了城乡低保标准自然增长机制和市区城乡低保一体化机制，并再次提高城乡低保标准。为全市1万名白内障患者免费实施了复明手术。为符合条件的低保家庭精神病患者和“三无”残疾人发放了生活救助金。新建、改扩建敬老院30所，农村五保对象集中供养率达到55%。全年累计开工建设经济适用房156万平方米，竣工103.7万平方米；廉租住房新开工15万平方米，建成2.04万平方米。人口和计划生育工作成绩显著，被评为全国流动人口计划生育服务管理先进单位、全国婚育新风进万家示范市，人口自然增长率控制在6‰以内。不断提高教育保障水平，全年累计开工建设18所中小学校，完成77所农村初中实验室装备任务，郑州师专升本已获国家评审验收通过，中央和省资金支持的11个中等职业教育项目全部开工建设。加强公共卫生基础设施建设，市六院、市七院和市疾控中心等项目主体完工，为全市96个乡镇卫生院各配备了1辆急救车，新建了1000个标准化村卫生所，做好手足口病、甲型H1N1流感等重大疫情的防控工作。大力实施文化惠民工程，新建了50个乡镇文化站和800个农村文化大院。认真实施全民健身工程，成功举办了市第十届运动会。

（十）民主法制建设和社会稳定工作得到加强。认真开展深入学习实践科学发展观、“讲党性修养、树良好作风、促科学发展”和大力弘扬焦裕禄精神等活动，持续推进“两转两提”，公务员队伍作风明显改进，综合素质不断提升，服务型政府建设迈出新步伐。主动接受人大及其常委会监督，支持政协履行职能，加强与各民主党派、工商联和无党派人士的联系沟通。办理省、市人大代表议案、建议和政协委员提案1155件，满意率和基本满意率达95%以上。高度重视政府法制工作，全年制定政府规章12件，提请市人大常委会审议地方性法规草案3件；加强行政执法责任追究，规范行政执法行为，提高行政执法效能。认真落实党风廉政建设责任制，政府廉政建设和反腐败工作取得新成效。加强精神文明建设，公民道德水平和城乡文明程度不断提升。严格落实安全生产责任制，对重点行业、重点领域进行全面检查和专项整治，安全生产形势总体平稳。深入开展“四严一创”，严厉打击各种违法犯罪行为，全力维护社会治安大局稳定。信访工作机制不断创新，应急管理体制不断完善。加强军政军民团结，双拥模范城创建取得新进展。

新闻出版、广播电视、统计、审计、侨务、对台事务、国防教育、民族宗教、社会科学、地方志、档案、气象和地震监测、人防等工作都取得了新的成绩。援助江油四镇灾后重建工作进展顺利。

回顾一年来的工作，在面对金融危机的严重影响下，我市经济社会能够实现又好又快发展，我们深刻体会到，得益于认真坚持和把握了以下几个方面：一是多策并举，科学应对，着力增强政策的针对性和实效性。认真贯彻落实中央、省扩内需、保增长一揽子政策措施，适时调整发展思路和工作重点，并及时研究出台我市应对金融危机的一系列政策措施，抓住开拓市场、服务企业、项目建设、招商引资等关键环节，扎实开展了企业服务年、产销对接、项目观摩、大招商等活动。二是长短结合，统筹兼顾，着力增强经济发展的后劲。辩证看待危机，处理好当前保增长与长远调结构促转型之间的关系，组织编制汽车、纺织服装、食品等产业发展专项规划，设立产业发展专项资金，力争危中求机、化危为机，为经济社会跨越式发展打牢基础。三是抢抓机遇，重点突破，着力打造经济发展的核心增长极。加快郑州新区的规划建设，努力把新区打造成中原城市群“三化”协调科学发展先导示范区、国家综合交通枢纽、全省经济社会发展的核心增长极。四是深化改革，扩大开放，着力增强经济发展的动力和活力。利用金融危机形成的“倒逼”机制，着眼于解决制约科学发展的突出矛盾和问题，深化国有企业改革，抓好重点领域改革，加快行政管理体制改革和投融资体制改革。抓住全球产业大转移、国内需求大流动的重大机遇，开展“南下北上”大招商活动，主动承接产业转移。五是以人为本，改善民生，着力促进经济社会和谐稳定发展。把维护群众根本利益作为工作的出发点和落脚点，围绕办好“十大实事”，加大公共支出，改善公共服务，解决了一大批人民群众最关心的利益问题。

各位代表，在罕见的困难和挑战面前，我市经济社会发展取得这样的成绩极其不易。这是省委、省政府和市委正确领导的结果，是市人大、市政协监督支持的结果，是全市广大干部群众共同奋斗的结果。在此，我代表市人民政府，向工作在全市各条战线的广大工人、农民、知识分子、干部、人民解放军指战员、武警官兵、公安民警以及各界人士致以崇高的敬意！向关心支持郑州发展的同志们、朋友们表示衷心的感谢！

在肯定成绩的同时，我们也清醒地认识到发展中存在的矛盾、困难和问题，主要表现在：发展方式还比较粗放，经济结构不尽合理，产业层次不高、竞争力不强等问题在危机中凸显，经济发展方式的转变刻不容缓；需求不足的约束尚未根本性改变，出口形势不容乐观，部分行业和企业生产经营依然比较困难，经济发展的内生动力还有待进一步增强；城市建设与管理需要进一步加强，交通拥堵成为急需解决的突出问题；农业发展的基础还不稳固，农业生产的抗风险能力还比较弱，农民增收还比较困难；一些政府工作人员思想观念陈旧、服务意识淡薄、工作作风不实、办事效率低下，影响了政府的公信力和执行力，服务型政府建设任重道远。对于这些问题，我们将保持清醒头脑，增强忧患意识，采取有力措施，切实加以解决。

二、2010年政府工作的总体要求和奋斗目标

2010年是实施“十一五”规划的最后一年，也是推进我市新一轮经济社会跨越式发展的关键之年，做好今年的各项工作具有十分重要的意义。

今年政府工作的总体要求：全面贯彻党的十七大和十七届三中、四中全会精神，以邓小平理论和“三个代表”重要思想为指导，深入贯彻落实科学发展观，继续大力实施跨越式发展战略，坚持重在持续、重在提升、重在统筹、重在为民，更加注重推动经济发展方式转变和经济结构调整，更加注重提高经济增长的质量和效益，更加注重推进改革开放和自主创新，更加注重改善民生、保持社会和谐稳定，更加注重利用国际国内两个市场、两种资源，着力推进工业化、城镇化、农业现代化协调发展，努力保持经济平稳较快增长和跨越式发展的良好局面。

今年经济社会发展的主要预期目标：生产总值增长13%；地方财政总收入增长11%，其中一般预算收入增长14%；全社会固定资产投资增长30%；社会消费品零售总额增长15%；市属及以下企业进出口增长12%；实际利用外商直接投资增长17%；居民消费价格涨幅控制在3%左右；城镇居民人均可支配收入和农民人均纯收入分别实际增长8%和9%；人口自然增长率控制在6‰以内；城镇新增就业13万人，城镇登记失业率控制在4%以内；单位生产总值能耗降低4.2%，化学需氧量和二氧化硫排放量分别下降3.87%和3.36%。

围绕上述要求和目标，今年政府工作将重点实施“一二三四五”的工作思路，即抓好一个载体、实施两个规划、建设三个体系、做强四大产业、打好五大战役。抓好一个载体，就是把产业集聚区建设作为科学发展的载体，解决好体制保障、要素支撑、项目落地等问题。实施两个规划，就是全面实施郑州市城市总体规划和郑州新区规划。建设三个体系，一是建设现代产业体系，制定完善现代产业体系发展规划，扶持发展战略支撑产业；二是建设现代城镇体系，统筹城乡发展，形成城乡一体发展的新局面；三是建设自主创新体系，通过培育主体、丰富载体、完善机制、抓好科技专项，推动产学研结合，增强产业核心竞争力。做强四大产业，就是做强汽车和装备制造、物流商贸、文化创意旅游和电子信息产业。打好五大战役，就是打好结构调整、郑州新区建设、重大项目建设、城市交通综合整治和新农村建设战役。

在具体工作中，我们将始终把握以下六个原则：一是始终把科学发展作为根本要求。以科学发展观为指导，全面推进经济、政治、文化、社会建设，促进现代化建设各个方面、各个环节相互协调，促进人与自然的和谐，实现经济社会永续发展。二是始终把结构调整作为工作主线。抓紧培育战略性支撑产业和主导产业，用先进适用技术改造提升传统产业，优化产业结构，构建现代产业体系。加大技术创新和技术改造力度，加大节能降耗和污染减排工作力度，发展循环经济，建设低碳城市。三是始终把郑州新区建设作为“一号工程”。进一步完善规划体系，推进管理体制机制创新，加大招商引资和项目建设力度，全面掀起新区建设高潮。四是始终把综合交通枢纽建设作为关键环节。进一步巩固和强化郑州的枢纽地位，加快建设铁路、公路、航空、信息“四港一体”的立体交通网络，打造内陆无水港，着力构筑现代大都市交通体系，通过构筑大交通，带动大物流，形成大产业，建设大都市。五是始终把投资和消费作为强劲动力。在优化投资结构的基础上，力争固定资产投资保持高速增长。提高消费能力，培育消费热点，增强消费对经济增长的拉动力量。坚定不移地实施开放带动战略，积极参与国际市场竞争，努力扩大出口份额。六是始终把改善民生作为最终目标。在决策规划、推进发展时以民为先，始终把人民群众的利益放在心上，从而赢得民心、集中民智、凝聚民力，形成发展的强大合力。

三、2010年政府工作的主要任务

2010年，市政府将紧紧围绕跨越式发展十大工程这一重要抓手和“一二三四五”的工作思路，把保增长、保态势、保民生、保稳定作为首要任务，立足当前、着眼长远，突出重点、统筹兼顾，确保全年各项目标任务圆满完成。

（一）推进经济结构调整，提高经济增长的质量和效益。全面实施产业调整振兴规划，着力推动制造业与服务业的融合、互动发展，加快构建现代产业体系。坚定不移地实施工业强市战略。制定出台并加快实施现代工业体系规划和汽车、装备制造业、电子信息、食品、纺织服装、新材料、铝及铝加工等专项规划，大力发展优势产业集群，全力打造一批在国内有影响的大企业和企业集团。以建设百万辆汽车生产基地为目标，依托郑州日产、宇通客车、海马（郑州）、少林四大整车企业，强力推进日产第二工厂一期工程、宇通客车新增2万台客车产能提升等重大项目建设，积极引进和推动中重卡汽车落户郑州；加快电动汽车产业配套设施体系建设，提升电动汽车整车研发和生产能力，推动电动汽车示范运营，创建电动汽车运营示范城市。依托大方桥梁、宇通重工等一批龙头企业和恒天集团新纺机、七一三所高科技产业园等重大项目，提高装备制造业的整体水平。积极发展电子信息产业，巩固提升光伏、新型电池产业，扩大绿色照明产业规模，提升电子元器件、信息安全、软件服务产业，加快推进中科电子信息产业研发基地等项目建设。以郑州纺织产业园服装生产基地、杉杉集团入驻、宏业纺织等项目为抓手，实施名牌战略，培育纺织服装创意产业。强力推进中粮（郑州）工业园、益海嘉里、双汇食品工业园、思念食品新工业园等项目建设，做大做强食品工业。加快中孚实业新建30万吨高档铝材生产线、宇通全铝车身公交车等项目。大力发展现代服务业。全面实施国际物流中心和商贸城建设规划，实施郑州出口加工区扩建工程，启动郑州国际物流园区、航空港综合物流园区基础设施建设，积极向国家申请设立航空综合保税区和B型保税中心，加快推进国家干线公路物流港、铁路集装箱中心站等物流园区建设，重点推动冷链物流、粮食物流、邮政物流等物流行业建设，争取郑州国际物流中心进入国家服务业综合改革试点。继续完善商业网点和流通体系，培育本土大型商贸流通企业集团，引进国内外知名的大型百货企业，鼓励并争取更多国际名牌入驻郑州。大力发展电子商务。组织实施《郑州区域性金融中心建设规划纲要》，大力发展金融机构，形成完善的金融体系，促进金融业快速发展；吸引国内外金融、期货、保险机构在郑设立总部，争取外资银行入驻郑州；支持郑州商品交易所创新期货衍生产品，推进化肥、甲醇等品种早日上市交易。加快地方金融机构发展，推进农村信用社改革，以县域为单位组建农商行、农合行，支持郑州银行跨区经营，积极发展村镇银行、贷款公司、资金互助组织和小额借款公司；大力发展金融中介机构，尤其是要建立农村产权流转交易机构和农村产权流转担保机构，激活农村经济发展活力。大力发展会展业，认真办好第十六届郑州全国商品交易会、第二届中国绿化博览会、第八届中国国际农产品交易博览会、中国农机产品订货交易会等重大展会。

（二）狠抓重大项目建设，拉动投资增长。把今年作为项目建设年，以跨越式发展十大工程重点项目为抓手，以招商引资项目落地为重点，主动开展工作，优化建设环境，推动项目建设，力争全年全社会固定资产投资突破3000亿元。加快基础设施项目建设。继续配合推进石武、郑徐铁路客运专线和连霍高速郑州至洛阳段扩建等工程，加快推进郑州至机场、郑州至开封、郑州至焦作城际轨道交通和郑汴物流通道工程，确保西绕城公路改扩建、京珠高速郑州至漯河段改扩建、中心区铁路跨线桥工程竣工投用，确保四环快速路运行通车，加快轨道交通1号线建设，开工建设轨道交通2号线，开工建设郑新快速通道、国道107线郑州段改建、航空港二期工程、综合交通枢纽公路客运站、南水北调供水配套、城镇供水设施扩能升级和管网改造工程，加快城市热力、天然气管网和设施的建设与改造步伐。强力推进重大产业发展项目，力争河南中烟150万大箱生产基地、恒天集团河南载货汽车基地、海马郑州零部件生产基地等13个已签约重大项目尽快开工建设，加快推进郑煤机高端液压支架、中船重工智能屏蔽门及电梯开发等一批在建重点工业项目，力争国电荥阳2×60万千瓦发电机组、旭飞液晶玻璃一期等76个项目按期达产。积极做好列入国家及省重点项目的推进工作，突出抓好宇通年产3万台悬架等47个省“8511”投资促进计划项目、郑州日产年产4万台多功能商用车等19个省“双百计划”项目和华润电力1×60万千瓦发电机组等154个省“1074”项目，确保全年完成投资285亿元。继续扩大民间投资，力争投资达到2000亿元。结合“十二五”规划和现代产业体系、自主创新体系、产业集聚区等一系列重大规划，再谋划、储备一批重大项目。实施产业集聚区投资倍增计划，力争完成投资700亿元，力争销售收入超百亿元的产业集聚区达到12个。优化项目建设环境，实行联审联批、政府投资项目代建制和社会投资项目代理制，坚决打击影响项目建设的不法行为。

（三）保持政策连续性，扩大消费需求。继续实施促进消费的政策措施，增强消费预期，优化消费环境，培育消费热点，增强消费对经济的拉动作用。加快建设一批重点商圈、特色商业街、社区便民店，支持大型百货、专业批发市场向二级城市布局网点。促进文化、旅游、信息、健身、家政、餐饮等服务消费。培育夜间消费，市内各区至少规划建设1条夜市步行街。全面实施放心早餐工程，方便市民生活。认真落实家电、汽车、摩托车下乡和家电、汽车以旧换新政策，增加农机具购置补贴，对农民购买国家规定品牌和型号的家电，按销售价格给予13%的直接补贴；将汽车以旧换新的补贴标准由3000~6000元提高到5000~18000元。继续实施新农村现代流通服务网络工程和“万村千乡”市场工程，新建100个农村连锁超市，覆盖全市90%的行政村。加强和改善房地产市场调控，促进房地产业健康发展。加大农村危房改造力度，鼓励农民建设新居。

（四）加强“三农”工作，大力发展都市农业和现代农业。继续实施强农惠农政策，落实好粮食直补、农资综合直补和农机具购置补贴，小麦、玉米、水稻等良种补贴以现金形式直补给农民，认真落实国家小麦、稻谷等粮油收购政策，进一步提高小麦最低收购价格。加快实施新一轮优势农产品区域布局规划，重点扶持蔬菜、水果、花卉等作物设施化生产，提高农业综合效益。完善农产品质量安全体系，提高农产品质量安全保障水平。实施《郑州市现代畜牧业发展规划》，促进畜牧业发展。加强农业科技推广和农业标准化体系建设，加快出口创汇农业发展，大力发展农民专业合作社。加强农业农村基础设施建设，重点加强农业综合生产能力建设，加快建设高标准农田，加强水利特别是中小型农田水利建设，全面完成大中型水库除险加固任务，完成杨桥灌区5万亩现代示范区建设工程。实施自来水村村通工程，使40万农民群众用上自来水。统筹安排县乡公路、村镇交通设施，新修改造农村公路500公里。新增农村户用沼气3万座、大中型沼气工程60处。新建132座村镇垃圾中转站，形成“组保洁、村收集、乡运输、县处理”的农村生活垃圾处理新机制。加快推进新农村建设，今年新农村建设的重点是推进新型农村社区和中心村建设，以村镇规划编制为契机，对现有村镇合理布局，引导农民向新型农村社区和中心村集中，支持有条件的县（市）区建设新农村示范区，率先实现城乡一体化。积极推进农村综合配套改革，稳定和完善农村基本经营制度，在依法自愿有偿的基础上，创新承包经营土地流转方式，培育土地银行、土地专业合作社、土地交易市场等中介组织，开展集体建设用地流转改革试点；整合各项涉农资金，提高资金的使用效率。深化集体林权制度改革，全面完成主体改革任务。继续做好南水北调工程郑州段的移民安置、征地拆迁等工作。

（五）扎实推进郑州新区建设，努力构建复合型新城区。郑州新区作为全省和全市的“一号工程”，今年将全面掀起开发建设高潮，年度投资达到410亿元以上，开发建设面积达到20平方公里。完善规划体系，争取郑汴新区总体规划尽快报请省人大审议通过，完成公共交通、文化旅游、市政基础设施、地下空间概念规划和住房建设等5个专项规划的编制。全力推动龙湖地区、高校和职教园区、航空港区等重点区域的开发建设，带动新区整体发展。加强交通和水电气暖等基础设施建设，全年完成投资205.3亿元。围绕项目建设，抓开工、抓进度、抓达产，全年新开工项目39个，投资132.2亿元。加大招商引资力度，争取中兴郑州产业园及研发中心、深圳华强科技文化产业基地、香港招商局集团开发及物流配送中心等重大项目尽快落地。加快管理体制和工作机制创新，实施投融资体制改革，充分发挥新区投融资平台作用，对土地实行一级垄断开发；加快新区基础设施建设企业债券发行工作，继续探索BT、BOT等开发建设模式；推进行政管理体制改革，完善大部门管理体制，探索新型公共管理模式。

（六）实施中心城市带动战略，积极构建现代化大都市。把卫星城建设作为今年城市建设与管理的重点，统筹协调城乡规划、土地利用总体规划和产业集聚区发展规划，加快卫星城基础设施和公共服务体系建设，加快卫星城与中心城区一体化进程。积极推进县域户籍制度改革，支持在城镇稳定就业和居住的农民有序转变为城镇居民，探索实行宅基地换住房和承包地换社保，加快农民向市民转变，逐步把卫星城发展成为人口在30万以上、城区面积在30平方公里以上的中等城市，构建大郑州都市圈。实施畅通工程，解决交通拥堵问题。强化规划的引导控制作用，按照“中心东移、功能外迁、降低密度、组团发展”的思路，调整优化城市空间布局，加快中心城区地处交通拥堵路段的机关事业单位和长途汽车站、物流市场的外迁步伐，合理疏散人流、车流。投资118亿元，大力开展以交通综合整治为重点的市政基础设施建设，力争交通状况有明显改善。建设北三环—中州大道和经三路立交等9项立交工程，实现三环路快速畅通；开工建设京广路—沙口路快速通道，做好陇海路高架快速路前期工作，构建市区交通“大十字”格局；开工建设一批下穿隧道工程。完善静态停车系统，在三环内建设公共停车场23处，设置停车泊位5360个。坚持建管并重，加强交通综合整治，提高规范化、智能化管理水平；加强市民文明出行教育，综合整治违章占道、违禁行驶、乱停车、随意横穿马路等不良行为，提升全社会文明交通意识；提倡绿色出行，优先发展公共交通；强化城市精细化管理，集中治理堵塞严重的路口、路段。加强对火车站和二七广场等地区的管理，进一步优化窗口形象。改造市区100条背街小巷，为30条无灯道路安装路灯；新增公厕50个，新增设公厕指向标志2000个；新建南郊热源厂1个，铺装供热管道10公里，新增供热面积50万平方米；新建改建天然气管道50公里，新发展用户8万户；新增城市绿地500万平方米，新植乔木50万株；扩建西流湖公园。加大城中村、旧城和棚户区改造力度，通过改造，控制城市建筑密度，增加开敞空间，利用地下空间，完善城市功能，提升城市品位。加快推进数字化城管体系建设，提升城市精细化管理水平。进一步加大城市违法占地、违法建设的整治力度。深入开展爱国卫生运动，增强人民群众的环境卫生意识。

（七）大力发展文化事业和文化产业，加快建设文化强市。繁荣发展文化事业，完成商城遗址公园一期、市图书馆新馆主体工程，启动商城遗址公园二期、黄帝故里二期、郑州市历史文化名城展示和西山古城保护工程，开工建设大河村遗址博物馆二期工程。继续实施文化惠民工程，新建改建社区文化活动中心100个、农村文化大院500个，培训基层文化骨干1000名。加快文化创意产业发展，推动创意动漫、数字影视、网络服务、服务外包等新兴文化产业的发展，引导扶持小樱桃动漫、华豫兄弟等文化企业扩大经营规模，着力打造国家动漫产业发展基地（河南基地）、中国郑州信息创意产业园等一批创意产业集聚区。繁荣哲学社会科学和文学艺术，大力扶持《小小说选刊》、《百花园》等文化品牌。加强文化市场建设与管理，培育文明健康的网络文化。加强重点文物和非物质文化遗产保护，推进嵩山历史建筑群申遗工作。加快发展旅游业，推动景区资源整合，深入推进与知名旅游企业战略合作，把嵩山景区打造成世界级旅游精品景区；按照国内一流标准，开发建设杜甫故里、北宋皇陵、新密古县衙、雁鸣湖等景区，使其成为推动旅游发展的新动力；加快旅游道路、游客集散中心、旅游标识等旅游基础设施建设，提升旅游服务水平。继续实施全民健身工程，开工建设市奥体中心、体育馆，为500个行政村建设“一场（标准篮球场）两台（两个室外乒乓球台）”，全市行政村“一场两台”建设任务全面完成。积极申办全国城市运动会，高标准举办第八届中国郑州国际少林武术节和第十八届中日韩青少年运动会。切实加强精神文明建设，加强社会主义核心价值体系建设，加强社会公德、职业道德、家庭美德、个人品德建设，加强诚信体系和未成年人思想道德建设，推动文明城市、文明单位、文明村镇等群众性精神文明创建活动蓬勃开展，不断提高公民文明素质和城乡文明程度。

（八）加快转变发展方式，增强综合竞争力和可持续发展能力。坚持在发展中促转变，在转变中谋发展，务求在一些重要领域和关键环节实现新突破。加快创新型城市建设，打造高技术产业基地，完善超硬材料、新能源汽车、盾构、生物医药、花卉等领域产业技术创新联盟，建设一批绿色新材料、环保产业、循环经济集聚区。强力推进落后产能淘汰和节能减排，严格控制水泥、电解铝、焦炭等高能耗产业的新增产能，加强耐材行业资源整合。实施节能产品惠民工程，加快推进“十城万盏”半导体照明应用试点工程，努力打造中西部重要的半导体照明产业基地。加强环境保护综合整治，市区和各县（市）城区的生活污水集中处理率分别达到90%和80%；加大机动车尾气排放和噪声污染治理力度，机动车尾气排放达标率和噪声污染查处率均达到80%；市区空气质量优良天数超过315天。加强林业生态市建设，新增造林18万亩，建成林业生态乡镇12个、林业生态村100个。加快生态水系建设，开工建设金水河上游、东风渠上游和潮河治理等项目，启动实施贾鲁河生态水系治理与河道复合型湿地建设工程，继续实施黄河湿地保护工程。

对地表水饮用水源地进行综合整治，全市饮用水源地水质达标率达到98%。完成各级土地利用规划修编，大力开展土地综合整治，将农村土地整治与城乡建设用地增减挂钩，定目标、定任务，优先保障重点项目和产业集聚区需求。

（九）深化改革开放，进一步增强发展的动力和活力。积极推进重点领域改革，为经济发展提供强大动力和体制保障。深化行政管理体制改革，按照省委、省政府批复的我市政府机构改革方案，3月底前全面完成市政府各部门“三定”工作。深化投融资体制改革，加强银企合作，加快中小企业投融资平台和信用担保体系建设；加快企业直接融资步伐，努力解决企业融资难问题；建立投融资平台运行监管机制，创新融资产品，拓宽融资渠道，防范融资风险。深化医药卫生体制改革，推进基本公共卫生服务均等化，全面开展公立医院改革试点，在全市乡镇卫生院和公办社区卫生服务中心推行国家基本药物制度，探索试行异地就医结算机制试点。深化文化体制改革，全面启动国有文艺院团转企改制，年底前完成市县两级国有院团转企改制任务；加快推进经营性报纸、期刊、出版单位转企改制，推进电台、电视台制播分离。深化企业改革，加快引进战略投资者，继续推进企业战略重组、兼并、收购工作；积极推进国有资本经营预算制度建设，发挥国有资本的调控、调节和导向作用；稳妥推进市属企业脱钩改制及经营性事业单位转企改制工作。大力发展非公有制经济，毫不动摇地鼓励、支持、引导非公有制经济的发展。

积极实施开放带动战略。持续有效地开展大招商活动，主动承接产业转移，争取设立承接转移示范区。依托产业集聚区建设，开展多种形式的招商活动。加强招商引资项目信息库建设，建立健全重大项目协调例会制度和进展情况督查通报制度。优化政务环境、法制环境和人文环境，着力打造一流的开放环境。抓住外部需求回暖的有利时机，落实鼓励出口的政策措施，重点支持传统优势产品、大宗农产品、自主品牌产品、自主技术出口和服务外包发展，努力实现出口恢复性增长。大力实施 “走出去”战略，不断增强配置全球资源的能力。加快航空口岸和东站货运口岸建设，推行“一站式”通关。

（十）切实保障和改善民生，全力维护社会稳定。加大民生投入，继续办好“十大实事”，认真解决好一批人民群众最关心的突出问题。继续实施积极的就业政策，以创业带动就业，强化对高校毕业生、失业下岗人员和农民工等就业困难群体的就业服务，加大小额担保贷款推进力度；继续实施“阳光工程”、“扶贫雨露”和农村劳动力技能培训计划，新增农村劳动力转移就业10万人、职业技能培训5万人。提高社会保障水平，完善城乡居民基本养老保险制度，在全市推广国家新型农村社会养老保险试点工作；提高企业退休人员基本养老金，人均每月增加120元；扩大城镇居民基本医疗保险覆盖面，提高城镇职工、城镇居民基本医保最高支付限额标准和住院支付比例；重视失地农民的生活问题，切实解决他们在就业、就学、社保、退伍安置等方面遇到的实际问题。加强社会救助，新建“留守儿童之家”和“流动儿童之家”30个；实施“阳光家园”计划，对符合居家托养条件的残疾人，每人每年补助1000元；对符合条件的残疾人托养服务机构，按托养人数每人每年补助3000元；高度关注居家养老工作，建立健全居家养老服务体系，形成政府购买服务和社会资源广泛参与的工作格局。加快保障性住房建设，新开工建设经济适用房150万平方米，竣工100万平方米；新开工建设廉租房15万平方米，竣工5万平方米；提高廉租住房廉租补贴发放标准，符合条件并申请廉租住房保障的家庭实现“应保尽保”。继续做好扶贫开发工作，完成扶贫搬迁1800户6500人。大力发展教育事业，市区新建、改扩建15所中小学校，加快推进公办幼儿园建设步伐；改善农村办学条件，为全市50所农村寄宿制中小学校配齐学生用床和餐用桌椅；实施职业教育攻坚计划，加大职业教育扶持力度，自今年秋季起对具有郑州户籍并在郑州市属及县（市）属中等职业学校就读的学生免除学费，实现中职生免费教育，整合重组市区职业教育资源，全面加快职教园区建设。加强公共卫生和重大医疗卫生设施建设，完善县、乡、村三级基层医疗卫生服务体系；全面推行新农合门诊统筹，将财政补助标准提高到每人每年120元，大病住院报销封顶线提高到6万元；加快市九院老年关爱病房楼、市儿童医院（东区）项目建设，改扩建市妇幼保健院，完成市七院迁建工程；新建796所标准化村卫生所，全面完成每个行政村都有一所标准化卫生所的任务，为新建村卫生所各配备2万元的基本医疗和健康教育设备；构建全市统一的紧急医疗救援体系，实现120急救报警网络全覆盖，新建7个市区急救站和67个乡镇卫生院急救站，为全市所有急救站各配备3辆专用急救摩托车。实施优生优育工程，免费开展出生缺陷一级干预，提高人口素质，改善人口结构，稳定低生育水平，引导人口与经济、社会、资源、环境协调发展。积极做好第六次全国人口普查工作。切实保障妇女和未成年人基本权益，大力推动慈善事业发展。深化平安建设，落实安全生产责任制，推动安全生产源头治理，严格实行煤矿安全监管制度，加大行政问责力度，严厉打击违法违规行为；进一步加强非煤矿山、化工、烟花爆竹、道路交通、消防等重点行业、重点领域的安全专项整治，坚决遏制重特大事故的发生。加强食品药品安全监管，继续开展食品药品安全专项整治。不断完善社会矛盾纠纷调处机制，认真解决损害群众利益的突出问题。高度重视并有效防范群体性事件和突发公共事件。严厉打击各类违法犯罪行为，切实维护社会大局的和谐稳定。

继续做好国防动员、人民防空工作，加强全民国防教育和民兵预备役建设，深入开展双拥共建活动。巩固平等、团结、互助、和谐的社会主义民族关系，发挥宗教界人士和信教群众在促进经济社会发展中的积极作用。充分发挥工会、共青团、妇联等人民团体的桥梁纽带作用。扎实做好统计、审计、外事、侨务、对台事务、地方志、档案、气象和地震监测等工作。继续做好江油四镇的对口援建工作。力争全面完成“十一五”规划的各项目标任务。编制好“十二五”规划。

四、切实加强政府自身建设

适应新时期经济社会发展的需要，以实施民生福利指标体系和科学发展评价体系为载体，扎实推进 “两转两提”，坚持科学性，增强紧迫性，突出实效性，切实加强政府自身建设，提高执行力，加大推动力。

（一）坚持依法行政。严格依照法定权限和程序管理经济社会事务。严格执行人大及其常委会的决议、决定，自觉接受人大及其常委会监督，及时报告工作；积极支持政协履行政治协商、民主监督、参政议政职能，充分听取各民主党派、工商联、无党派人士及各人民团体的意见和建议。切实办好人大议案、建议和政协提案。广泛接受社会公众和新闻舆论监督，强化监察、审计等专门监督。依法推进政务公开，切实保障人民群众的知情权、参与权、表达权和监督权。严格执行行政问责制，提高政府执行力和公信力。健全依法行政评议考核制度，规范行政执法行为。

（二）加快职能转变。更好地履行经济调节和市场监管职能。加快推进政企分开、政资分开、政事分开、政府与市场中介组织分开；推进公平准入，加强对涉及人民群众生命财产安全领域的监管，特别是把涉及人民群众身体健康和生命安全的食品、药品等作为监管重点。更加注重社会管理和公共服务。强化政府促进就业和调节收入分配职能，完善社会保障体系；健全基层社会管理体制，加强社区建设，发展社会工作者队伍，完善政府、社会组织和公民之间的公共治理机制；建立健全公共服务体系，稳步增加政府公共服务支出，提高公共服务的质量和水平。深化行政审批制度改革，进一步清理、减少和规范行政审批事项。

（三）转变工作作风。把求真务实的要求落实到政府工作的各个环节，一心一意谋发展，集中精力抓落实，克服官僚主义，防止急功近利。坚持改进会风、文风，严格控制和规范各种检查、评比、达标活动，减少不必要的应酬和事务性活动。深入调查研究，密切联系群众，关心群众疾苦，倾听群众呼声，为群众排忧解难。厉行节约，反对浪费，建设节约型机关。加强公务员队伍能力和作风建设，建立健全公务员考核评价、激励约束机制，深入开展人民满意公务员创建活动，切实解决少数公务员服务意识不强、办事效率不高等突出问题。

（四）加强廉政建设和反腐败工作。深入推进惩治和预防腐败体系建设。完善工程招投标、土地使用权出让、政府采购等制度，规范公共资源交易行为。继续开展工程建设等领域的专项治理工作。坚决查处腐败案件，严肃查处权钱交易、失职渎职、侵害群众利益等违纪违法行为。严格落实党风廉政建设责任制，严格执行领导干部廉洁自律各项规定，勤俭办事，秉公用权，树立各级政府和公务员公道正派、廉洁为民的新形象。

各位代表！

郑州是全省的重心所在，郑州的发展事关中原崛起，郑州的形象事关河南的形象。我们必须增强省会意识、大局意识、责任意识，积极发挥辐射带动作用，力争各项工作走在全省的前列。

各位代表！

新的一年，郑州的发展蕴含着更大的机遇，同时也面临着更加严峻的挑战。让我们紧密团结在以胡锦涛同志为总书记的党中央周围，在省委、省政府和市委的坚强领导下，深入贯彻落实科学发展观，解放思想，开拓创新，真抓实干，勇争一流，为实现郑州经济社会跨越式发展、为助推中原崛起而努力奋斗！
